# Grouting Mechanism of Polyurethane Composite Materials in Asphalt Pavement Subsidence

**DOI:** 10.3390/ma16217052

**Published:** 2023-11-06

**Authors:** Maoping Ran, Xinxing Zhou, Yuan Yan, Ruiqie Jiang, Xinglin Zhou

**Affiliations:** 1 School of Automotive and Traffic Engineering, Wuhan University of Science and Technology, Wuhan 430081, China; ranmaoping@wust.edu.cn; 2 Institute of Resources and Environmental Engineering, Shanxi University, Taiyuan 030006, China; zxx09432338@sxu.edu.cn; 3 School of Urban Construction, Wuhan University of Science and Technology, Wuhan 430070, China; yanyuan@wust.edu.cn; 4 School of Machinery and Automation, Wuhan University of Science and Technology, Wuhan 430081, China; jiangruiqie@wust.edu.cn

**Keywords:** asphalt pavement, subsidence, grouting mechanism, performance evaluation, polyurethane composite material

## Abstract

The mechanical properties of polyurethane grouting materials were significantly improved when cement, sodium meta-silicate, red mud, slag, and fly ash were added. However, the grouting mechanisms of polyurethane composite materials are not clear. The grouting mechanisms of polyurethane composite materials in asphalt pavement subsidence were investigated. The results of computed tomography analysis show that polyurethane foam is filled with geopolymer hydration products. The results from ground penetrating radar after grouting show that mapping has no significant fluctuation or dislocation effect, which indicates that the grouting effect is strong. The high-density electrometer can also test the pavement subsidence place and distribution. The grouting mechanisms indicate that polyurethane foam acts as the consolidation structure, and the geopolymer filled with the foam pores of polyurethane and geopolymer forms a stable consolidated body. The seriflux includes under-layer seriflux (red mud, slag, water, and polyurethane composite materials) and upper-layer seriflux (polyurethane seriflux), and there exists a weak phase separation phenomenon, in which the separation phase is mainly polyurethane with little red mud-based geopolymer.

## 1. Introduction

By the end of 2021, China’s highway maintenance mileage was 5.25 million kilometers and accounted for 86.3% of asphalt pavement [1,2,3]. The recessive disease of asphalt pavement has become a key factor restricting the development of highway use quality and maintenance quality in China [4]. As a new type of grouting material for trench-less grouting treatment of hidden diseases of asphalt pavement, polyurethane has remarkable advantages such as rapid expansion, low weight, and good mechanical properties [5,6]. Polyurethane grouting material is made of isocyanate, polyether polyol, catalyst, chain extender, foaming agent, defoaming agent, and other raw materials, and belongs to the chemical grouting material category [7,8]. Those working with polyurethane grouting material can easily control the form and performance of a product by changing the type and share of its raw materials, so as to obtain the hard polyurethane foam [9,10]. Furthermore, the setting and curing time of polyurethane can be controlled comprehensively by adjusting the catalyst content and proportion of polyether polyol and isocyanate in polyurethane raw materials [11,12]. Reducing the curing time can realize rapid opening to traffic after road maintenance. Cement or other cementitious materials are added into polyurethane, which makes the strength and durability of polyurethane composite materials increase [13]. Furthermore, the addition of cement or other cementitious materials (geopolymer) can also reduce the cost of grouting materials. So, polyurethane composite materials will become the optimization selection of grouting material for asphalt pavement subsidence. Research found that the density was the main factor affecting the compression strength of polyurethane composite materials. The lower the density of the polyurethane composite material, the better it performed under the condition of ensuring the strength of the polyurethane composite material. Moreover, there was a functional relationship between the stress level and fatigue life of polyurethane composite materials [5]. In theory, the greater the fatigue life, the better, which is achieved with a low stress level. The polyurethane chain segments resist the cyclic loading through the chain segment motion during the polyurethane curing process. The main reason for structure adjustment is the micro-phase separation, and the hydrogen bond breakage is the main source of energy dissipation [11]. The above research aimed to state that polyurethane composite materials are promising materials for asphalt pavement subsidence grouting. However, the grouting mechanisms of polyurethane composite materials in asphalt pavement subsidence are unknown, which has limited the application of polyurethane composite materials.

In recent years, the grouting technology of polyurethane composite materials has developed rapidly, such as with the grouting of shallow disease [14,15]. As for the shallow disease detection of asphalt pavement, ground penetrating radar can be used, but deep water-rich detection technology needs to be further studied [16,17]. The high-density electrical method is used to test deep disease and the test depth can reach up to 35 m [18]. The above research aimed to state that the new materials can affect significantly the detecting precision of the high-density electrical method and ground penetrating radar. However, how to use ground penetrating radar and a high-density electrometer to evaluate the grouting effect after polyurethane composite materials’ grouting deserves our exploration and study. Researchers modified asphalt by adding polymers to improve the road performance of asphalt [19,20,21]. After the use of rubber as an asphalt modifier, more and more polymers have been used in the field of modified asphalt [22,23,24,25] whose properties are different, and their modification effects on asphalt are different.

In this study, aspects of the performance such as density, compressive strength, the permeability coefficient, and the pore volume fraction of polyurethane composite materials’ consolidated core samples were evaluated. The grouting effect and mechanisms of polyurethane composite materials were investigated by ground penetrating radar, the high-density electrical method, computed tomography, and a scanning electron microscope.

## 2. Materials and Methods

### 2.1. Raw Materials

Poly-methylene poly-phenyl poly-isocyanate (PAPI), 1,2-Ethanediamine (1,2-E), and aromatic polyester (AP) were provided by Dow Chemical Co., Ltd. (Midland, MI, USA). n-Butane (n-B), 1,4-Butanediol (1,4-B), triethanolamine (TL), and silicone oil (SO) were bought from Shanghai Macklin Biochemical Co., Ltd. (Shanghai, China). Red mud (RM) came from Shanxi Yangquan Aluminum Industry Co., Ltd. (Yangquan, China), and its main components are Al_2_O_3_, SiO_2_, CaO (the mass ratio is 25.03:22.10:15.77). Slag selected was blast furnace slag (BFS), which came from Shanxi Taigang Stainless Steel Co., Ltd. (Taiyuan, China). The main components of the slag are CaO (40%), SiO_2_ (35%), and Al_2_O_3_ (15%). Sodium meta-silicate modules are 3.3, and they act as an alkali activator. The RM and BFS were the additives to the polyurethane grouting materials. The specific composition of the polyurethane composite materials is shown in Table 1.

### 2.2. Preparation of Polyurethane Composite Material

RM, BFS, and sodium meta-silicate were mixed with a glass rod in a 1000 mL beaker at first. The mix time selected was 30 min with hand stirring. Then, we added the 1,2-E, AP, n-B, 1,4-B, TL, and SO into the beaker with hand stirring for 15 min. Finally, PAPI was added to prepare the polyurethane composite materials with hand stirring for 2 min. The preparation process of polyurethane composite material was finished.

### 2.3. Nondestructive Testing of Asphalt Pavement Subsidence

The nondestructive testing of asphalt pavement subsidence used ground penetrating radar (SIR-3000, Geophysical Survey Systems, Inc., Nashua, NH, USA) and a high-density electrometer (WGMD-9, Chongqing Benteng Digital Control Technical Institute, Chongqing, China). The speed of the ground penetrating radar was 10 km/h. The car-right-wheel track of the lane was detected. The test parameters of ground penetrating radar included the following: the center frequency of the antenna was 100 MHz, the time window was 200 ns, and the detection depth was about 10 m. The test parameters of high-density electrometer included the following: we used the distributed measurement method, the total number of conversion electrodes was 110, and the pole distance was 2 m. The maximum isolation coefficient was 7. The inversion error was 9.4% and the number of iterations was 10. The pavement structure included two layers of asphalt mixtures: 4 cm AC-13 asphalt mixtures (SBS asphalt) and 6 cm AC-20 asphalt mixtures (SBS asphalt). This highway belongs to a heavily trafficked road.

### 2.4. Performance Tests of Polyurethane Composite Materials

The compressive strength and density test of polyurethane composite materials used the cube sample with a size of 40 mm × 40 mm × 40 mm. The density was measured according to the underwater weight method and JTG E20-2011 [26]. The compressive strength was measured at the loading speed of 0.5 MPa/s. The setting time of polyurethane composite materials was measured according to JC/T 2041-2020 (Polyurethane Grouting Material) [27]. The permeability coefficient of polyurethane composite materials was tested according to GB/T50123-1999 (Geotechnical Test Method Standard) [28].

### 2.5. The Grouting Effect Tests of Polyurethane Composite Materials

The grouting effect was tested by ground penetrating radar, the high-density electrical method, computed tomography (CT, General Electric), and scanning electron microscope (SEM, Carl Zeiss AG, Oberkochen, Germany). For CT, we used a GE Vtomex, and its parameters included 120 kV scanning voltage, 150 μA current, and 63.9 microns resolution. The sample appeared as a cylinder, and the height and width of the CT sample were 5 cm and 1.5 cm, respectively. The pore volume fraction was measured by CT. For an SEM, we used the ZEISS Sigma 300 to test the micro-morphology of the polyurethane composite materials’ consolidated core samples with 0.02–30 kV accelerating voltage, and 3 pA~20 nA for the probe beam. For Energy Disperse Spectroscopy (EDS), we used OXFORD Xplore (Aachen, Germany) with point scanning. The SEM sample appeared as a bulk and it was selected from the cylinder. The bulk sample needed to be sprayed with gold.

## 3. Results and Discussion

### 3.1. The Nondestructive Evaluation of Asphalt Pavement Subsidence

**Figure 1:**
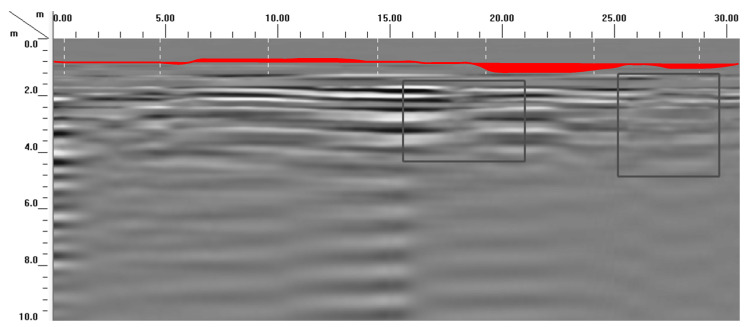
The picture of ground penetrating radar before grouting.

As shown in Figure 1, the radar electromagnetic wave reflection is strong, and the reflection interface is clear, which indicates that the selected road segment exhibits subsidence and a cavity under the road surface of 2~4 m. The red line separates the asphalt pavement layer from the foundation. It indicates that the asphalt pavement thickness is 1.04 m, which is bigger than that of conventional asphalt pavement thickness (0.18 m). The reason is that owing to the asphalt pavement subsidence, the asphalt pavement has been overlaid many times. The subsidence and cavity are reflected in the wavy peak valley in the ground penetrating radar picture. Furthermore, the cavity place is reflected in the partial shadow cavity in the ground penetrating radar picture. It indicates that ground penetrating radar is a good method to test the pavement subsidence.

**Figure 2:**
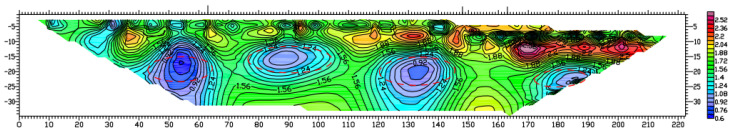
The picture from the high-density electrometer before grouting.

As shown in Figure 2, the results from the high-density electrometer show that the overall change in the resistivity value is complicated; the horizontal fluctuation of resistivity values is low, which is presumed to be qua-ternary strata. The red dotted line is the soft foundation under the road surface of 10~25 m, where the resistivity value is the smallest. There exists a cavity above the soft foundation. Furthermore, the resistivity value of the soft foundation is very close to the resistivity value of water, which indicates that there may exist water in the soft foundation areas. The results from the high-density electrometer also show that there is a big difference in the resistivity value when the test place is different. It indicates that there exist big differences in geology at the different test places and the high-density electrometer can also test the pavement subsidence place and distribution.

### 3.2. The Grouting Design of Polyurethane Composite Materials

**Figure 3:**
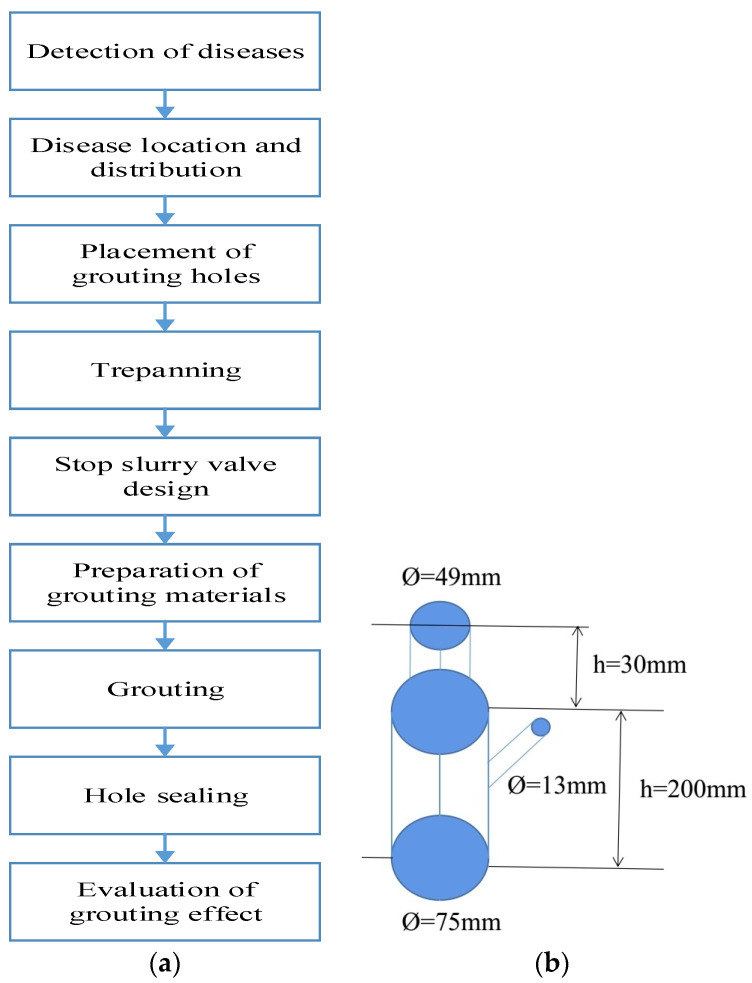
The grouting design of polyurethane composite materials: (**a**) the grouting roadmap of polyurethane composite materials, (**b**) **design schematic diagram of stop valve for double liquid grouting method**.

As shown in Figure 3a, the grouting design of asphalt pavement subsidence included the detection of road surface diseases, determination of disease location and distribution, placement of grouting holes, trepanning, stop slurry valve design, preparation of polyurethane composite materials, hole sealing, and evaluation of grouting effect [29]. Owing to the fast setting time of polyurethane, we designed this stop slurry valve. The design of the stop slurry valve is very important for the grouting, and the stop slurry valve is shown in Figure 3b. Polyurethane seriflux was injected from the 13 mm hole and geopolymer seriflux was injected from the 49 mm hole.

### 3.3. The Grouting Construction Case of Polyurethane Composite Materials

**Figure 4:**
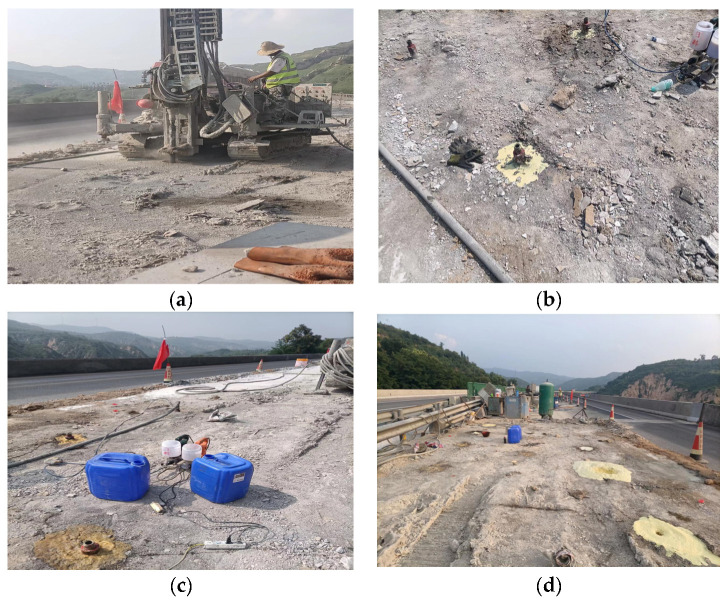
Schematic diagram of grouting construction process: (**a**) trepanning, (**b**) installing the hole sealer, (**c**) grouting, (**d**) hole sealing.

As shown in Figure 4, the grouting construction progress showed that the grouting construction case included trepanning, installing the hole sealer, grouting, and hole sealing. Polyurethane composite material is not only suitable with shallow layer grouting, but is also suitable with deep grouting. Owing to the low content of polyurethane, the cost of polyurethane composite material is much lower than pure polyurethane grouting materials. And the mechanical properties and waterproofing capability of polyurethane composite materials are better than that of pure polyurethane grouting materials and geopolymer grouting materials [30,31,32,33]. Of course, the cost of polyurethane composite material is larger than that of geopolymer grouting materials. The cost of polyurethane composite material is about 1950 RMB/t, the cost of polyurethane is about 15,000 RMB/t, the cost of geopolymer is about 500 RMB/t. So, the cost of polyurethane composite material falls in between that of geopolymer and polyurethane. However, the performance or grouting effect of polyurethane composite material is better than that of geopolymer or polyurethane grouting materials [33], because geopolymer fills with the foam pores of polyurethane and improves the foam strength. Due to their low cost and comprehensive economic benefit, especially for pavement subsidence grouting in rich water areas, polyurethane composite material is a good selection.

### 3.4. The Fundamental Properties Evaluation of Polyurethane Composite Materials

As shown in Table 2, the fundamental performance of polyurethane composite materials’ core samples showed that as the red mud content increased, the density, compressive strength, and setting time of polyurethane composite materials increased. The total mass was certain and the red mud content increased, while the blast furnace slag content decreased. As we well know, red mud mass is bigger than that of blast furnace slag. So, the compressive strength of polyurethane composite materials’ core samples increases with the increase of red mud content. However, the permeability coefficient of polyurethane composite materials decreased with the increase in red mud content. It indicated that red mud content can affect significantly the fundamental properties of polyurethane composite materials. And red mud can reinforce the mechanical property of polyurethane grouting materials, while weakening the waterproofness of polyurethane grouting materials.

### 3.5. The Grouting Effect Evaluation of Polyurethane Composite Materials

**Figure 5:**
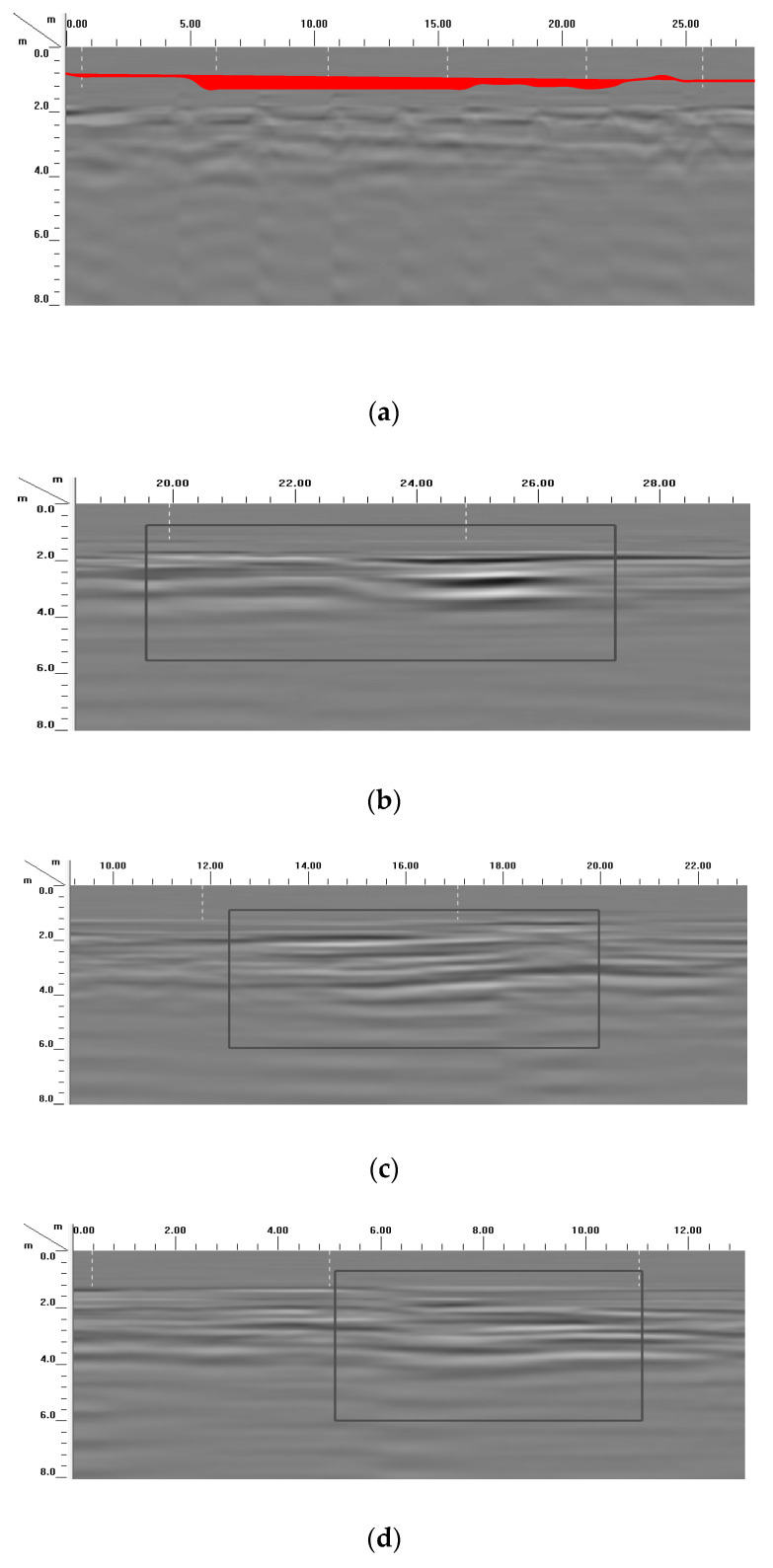

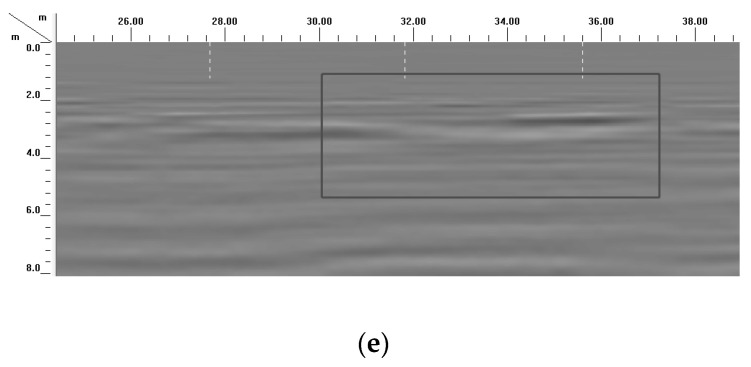
The picture of ground penetrating radar after grouting: (**a**) 5#, (**b**) 4#, (**c**) 3#, (**d**) 2#, (**e**) 1#.

As shown in Figure 5a, the results show that the radar electromagnetic wave reflection appears flat without a wavy peak valley. There did not exist characteristic peaks of the radar electromagnetic wave reflection picture ranging from the depth of 0 m to 10 m. And, the original strong wave peaks disappeared. The results showed the polyurethane composite materials (sample 5#) can repair the pavement subsidence. The grouting effect of the polyurethane composite material is good for the treatment of pavement subsidence. This results are the same as those from the previous research [1]. As shown in Figure 5b–e, there were some radar electromagnetic reflection waves. This indicated that the grouting effects of the polyurethane composite materials (samples 1#–4#) are not good.

**Figure 6:**
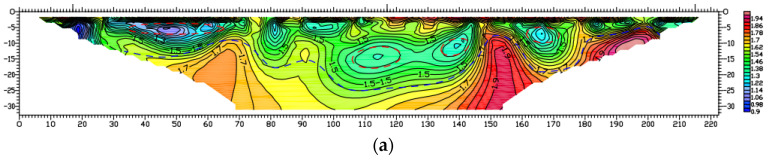

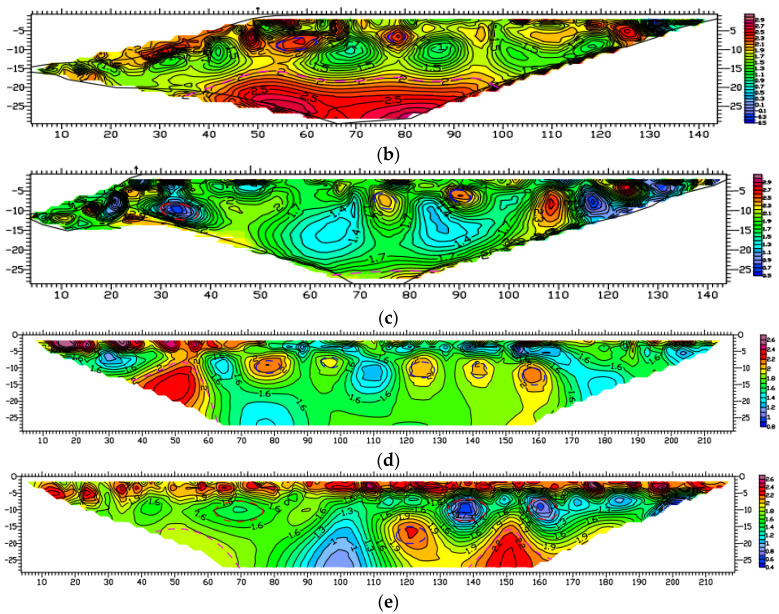
The picture from the high-density electrometer after grouting: (**a**) 5#, (**b**) 4#, (**c**) 3#, (**d**) 2#, (**e**) 1#.

As shown in Figure 6a, the high-density electrometer picture after grouting indicates that the resistivity shows a high and low variation trend, which is a typical H-type geoelectric layer structure [23]. The original subsidence and cavity areas were filled with polyurethane composite materials (sample 5#) and the resistivity of the original subsidence areas increased. The road surface had some low resistivity areas, which showed that the polyurethane composite grouting material drains the moisture of the roadbed above the grouting hole. Moreover, polyurethane composite grouting material itself is a kind of high-resistivity material. These results are in good agreement with the results of core sampling. As shown in Figure 6b–e, there are some diseases in these grouting areas. So, we can conclude that the grouting effects of samples 1#–4# are not good. The grouting effects of sample 5# indicate that it is the best for asphalt pavement subsidence.

**Figure 7:**
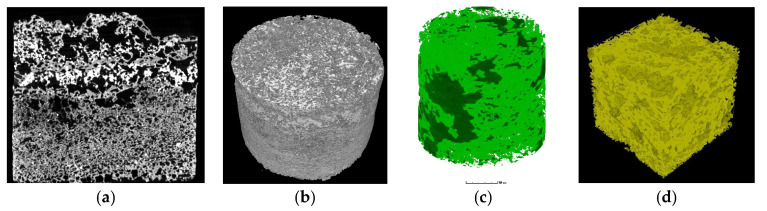
CT of polyurethane composite material (sample 5#) consolidated core samples: (**a**) cross section, (**b**) three dimensional top view, (**c**) three dimensional color view, (**d**) the inner view of core sample.

As shown in Figure 7a, the computed tomography of the polyurethane composite materials’ core samples shows that the pore volume fraction of the polyurethane composite materials’ core sample is 22.5%. The majority of pores appear in the upper layer of the polyurethane composite materials’ core sample, and there are few pores in the under-layer of the polyurethane composite materials’ core sample. As shown in Figure 7b, on the whole, the core compactness of the polyurethane composite grouting material is better and the grouting effect is better. As shown in Figure 7c, where the green area represents polyurethane and the black area represents geopolymer, polyurethane was distributed at the most areas in the core samples, whereas geopolymer was only distributed at the pore areas in the core samples. As shown in Figure 7d, the inner part of a core sample is close-grained, and foam pores are filled with red mud-based geopolymer. Usually, polyurethane foam acts as the skeleton structure and the red mud-based geopolymer will be filled with the foam pores of polyurethane; polyurethane and red mud-based geopolymer form a stable consolidated body.

**Figure 8:**
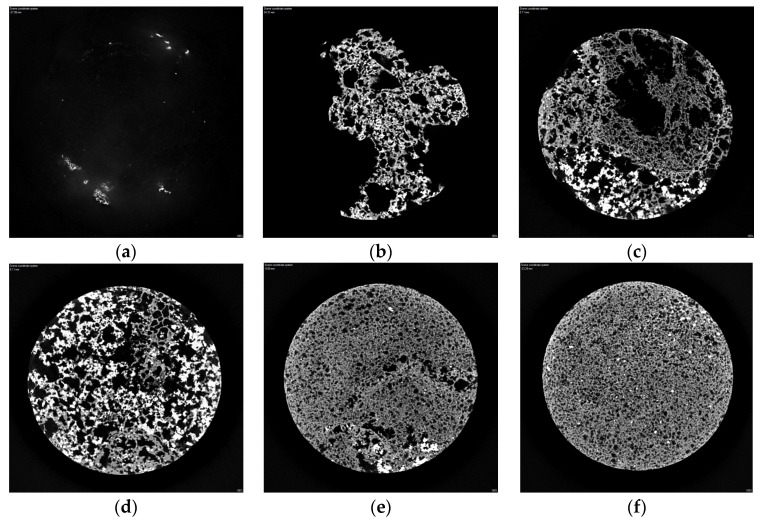
The vertical section CT of polyurethane composite materials’ (sample 5#) core sample: (**a**) surface 0 cm, (**b**) 1 cm depth, (**c**) 2 cm depth, (**d**) 3 cm depth, (**e**) 4 cm depth, (**f**) 5 cm depth.

As shown in Figure 8, the height of the core sample is about 6 cm, and at 1 cm deep the core sample has a vertical section; the vertical section of the core sample shows that the upper layer or surface of the core sample could not be filled with polyurethane composite materials. As with the depth increase of the vertical section, the polyurethane content increased firstly, then decreased. It indicated that owing to the density difference between polyurethane and red mud, the polyurethane composite materials are not uniformly distributed throughout the system in the core sample. So, as much as possible, care should be taken to make polyurethane and red mud mix evenly in the process of mixing. The storage stability of polyurethane composite slurry should be attended to.

**Figure 9:**
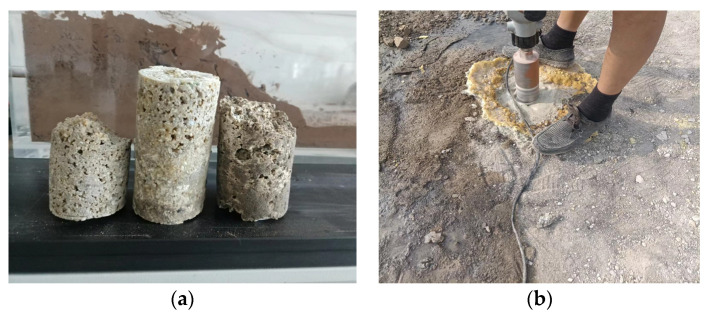
The polyurethane composite materials’ (sample 5#) consolidated core samples: (**a**) core samples, (**b**) drilling the core.

As shown in Figure 9a, the polyurethane composite materials’ core sample shows that owing to the existence of water and carbon dioxide emission, the upper layer porosity of the polyurethane composite materials’ core sample is more than that of the under-layer of the polyurethane composite materials’ core sample. Moreover, the external layer’s porosity of the core sample is more than that of the inner layer of the polyurethane composite materials’ core sample. As shown in Figure 9b, the water cannot immerse into the polyurethane composite materials, and always stays on the surface of the polyurethane composite material during the process of drilling the core, which indicates that polyurethane composite materials have good waterproofing capability and polyurethane composite materials’ grouting can get a good waterproofing effect.

**Figure 10:**
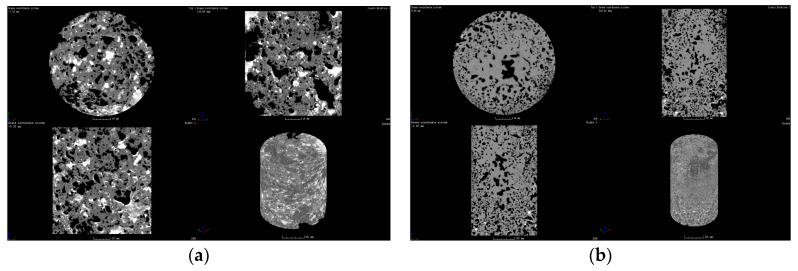
The CT of polyurethane composite materials’ (sample 5#) consolidated core samples: (**a**) upper layer, (**b**) lower layer.

As shown in Figure 10, the CT shows that the porosity of the upper layer of the polyurethane composite materials’ consolidated core sample is greater than that of the lower layer. There are some big pores in the upper layer of the polyurethane composite materials’ consolidated core sample, which indicates that the pores of polyurethane foam are not filled with geopolymer hydration products and the mechanical properties of the polyurethane composite materials’ consolidated core sample are not at their best. Owing to the compaction and diffusion of the polyurethane composite materials with high grouting pressure, there are some small pores in the lower layer of the polyurethane composite materials’ consolidated core sample, which indicates that the porosity of the polyurethane foam is filled with geopolymer hydration products and the mechanical properties of the polyurethane composite materials’ consolidated core sample are at their best.

**Figure 11:**
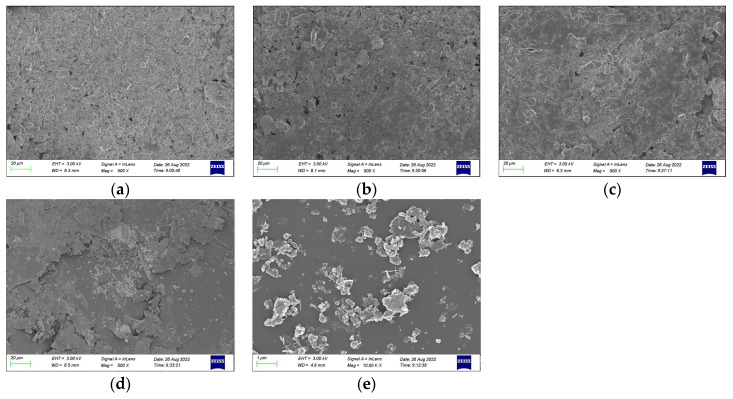
SEM image of polyurethane composite materials’ consolidated core samples: (**a**) 1#, (**b**) 2#, (**c**) 3#, (**d**) 4#, (**e**) 5#.

As shown in Figure 11, use of the scanning electron microscope (SEM) on a core sample shows that the polyurethane composite materials consist of many closed pores and few open pores. As with the increase in red mud contents, the open pores decreased, and we could not find the pores in the 5# sample, which indicates that the structure density of the core sample increases with the increase in red mud contents. The pores are very uniform and the foam size is very concentrated. The pores uniformly increased with the increase in the addition of red mud contents. As with the increase in red mud contents, the inner foam filled with red mud-based geopolymer and open pores decreased. It indicated that red mud content can affect significantly the pore uniformity and micro-morphology of the polyurethane composite materials’ consolidated core samples.

**Figure 12:**
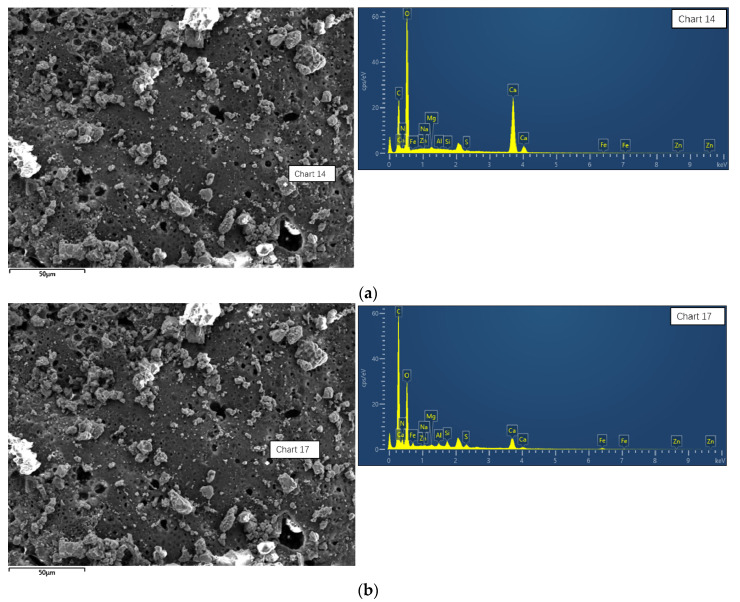
Energy spectrum of polyurethane composite materials’ (sample 5#) consolidated core samples: (**a**) point 1, (**b**) point 2.

As shown in Figure 12, the 5# energy spectrum (EDS) results showed that there is much C, O, N, Ca, Mg, Si, and Al. The C, O, and N belong to the polyurethane and Ca, Mg, Si, and Al belong to red mud-based geopolymer. Semi-quantitative calculation finds that there is 35% geopolymer and 65% polyurethane in point 1. And there is 10% geopolymer and 90% polyurethane in point 2. Compared with the results of area 1 and area 2, the contents of the geopolymer and polyurethane are different, and the difference is very big. Owing to the fact that the geopolymer/polyurethane ratio is 85:15, the EDS results are not suitable, which demonstrates that the upper core sample is usually filled with polyurethane rather than red mud-based geopolymer and there is in-homogeneous distribution of polyurethane composite materials. The content of polyurethane is greater in the polyurethane composite materials’ core sample. Further, polyurethane and red mud-based geopolymer are alternated in selected areas and the geopolymer is dispersed in the pores of the polyurethane foam.

### 3.6. The Grouting Mechanisms of Polyurethane Composite Materials

**Figure 13:**
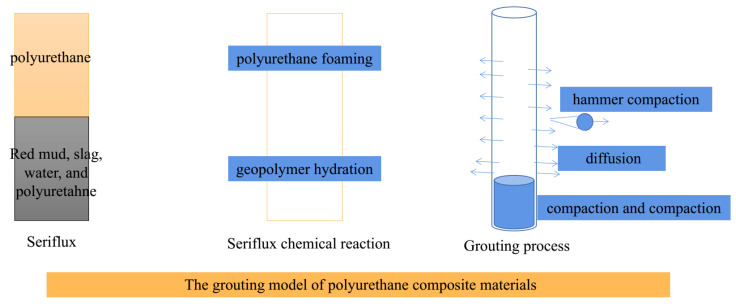
The grouting model of polyurethane composite materials.

As shown in Figure 13, the grouting mechanisms model of polyurethane composite materials shows that seriflux was divided into two layers: the under-layer is red mud, slag, water, and polyurethane composite materials seriflux; the upper layer is polyurethane seriflux, and there exist few phase separation phenomena, which is why the separation phase is mainly polyurethane. The seriflux chemical reaction was divided into two reactions, geopolymer hydration and polyurethane foaming, and geopolymer hydration products fill the pores of polyurethane foam. The grouting process can be divided into three steps: compaction and the compaction process of polyurethane composite materials, the diffusion process of polyurethane composite materials, and the hammer compaction process of polyurethane composite materials. The grouting mechanisms of polyurethane composite materials show that the slurry consolidation body can be divided into three layers: the polyurethane foam layer, the polyurethane–geopolymer composite layer, and the geopolymer layer. Of course, the geopolymer layer also includes some polyurethane, only the content is very little.

## 4. Conclusions

The grouting mechanisms and performance of polyurethane composite materials in asphalt pavement subsidence were investigated. We can draw the following conclusions:

The density of polyurethane composite materials is greater than that of polyurethane and it has a micro-foaming function. The compressive strength of the grouting consolidated core sample can reach up to 52.1 MPa and the minimum permeability coefficient of the grouting consolidated core sample is 1.0 × 10^−7^ cm/s.

Ground penetrating radar is a good method to test pavement subsidence, and there exist big differences in geology in the different test places, and a high-density electrometer can also test the pavement subsidence place and distribution. Polyurethane composite materials can act as seriflux materials and repair the pavement subsidence. The grouting effect of polyurethane composite material is good.

Usually, polyurethane foam acts as the consolidation structure, and the geopolymer filled with the foam pores of polyurethane and geopolymer forms a stable consolidated body. Owing to the density difference between polyurethane and geopolymer, the polyurethane composite materials are not uniformly distributed throughout the system in the core sample.

The seriflux chemical reaction was divided into two reactions, geopolymer hydration and polyurethane foaming, and geopolymer hydration products fill the pores of polyurethane foam. The grouting process can be divided into three steps: the compaction and compaction process of polyurethane composite materials, the diffusion process of polyurethane composite materials, and the hammer compaction process of polyurethane composite materials.

Owing to the high cost of polyurethane materials, low-cost and high-performance polyurethane composite materials should be developed, especially for low-content polyurethane and high-content solid waste.

## Figures and Tables

**Table 1:** The specific composition of polyurethane composite materials (**mass, g**).

Sample ID	PAPI	1,2-E	AP	n-B	1,4-B	TL	SO	RM	BFS
**1#**	**20**	**20**	**25**	**2**	**2**	**0.2**	**0.8**	**30**	**0**
**2#**	**20**	**20**	**25**	**2**	**2**	**0.2**	**0.8**	**20**	**10**
**3#**	**20**	**20**	**25**	**2**	**2**	**0.2**	**0.8**	**10**	**20**
**4#**	**20**	**20**	**25**	**2**	**2**	**0.2**	**0.8**	**5**	**25**
**5#**	**20**	**20**	**25**	**2**	**2**	**0.2**	**0.8**	**0**	**30**

Remaks: PAPI-Poly-methylene poly-phenyl poly-isocyanate, 1,2-E-1,2-Ethanediamine, AP—aromatic polyester, n-B—n-Butane, 1,4-B—1,4-Butanediol, TL—triethanolamine, SO—silicone oil, RM—red mud, BFS—blast furnace slag.

**Table 2:** The fundamental performance of polyurethane composite materials.

Sample ID	Density (g/cm^3^)	Compressive Strength (MPa)	Setting Time (s)	Permeability Coefficient (cm/s)
**1#**	**0.825**	**52.1**	**1800**	**1.2 × 10^−8^**
**2#**	**0.756**	**49.8**	**1200**	**4.3 × 10^−8^**
**3#**	**0.713**	**42.5**	**900**	**8.9 × 10^−8^**
**4#**	**0.707**	**38.2**	**750**	**9.6 × 10^−8^**
**5#**	**0.528**	**25.0**	**300**	**1.0 × 10^−7^**
**Geopolymer**	**2.782**	**42.3**	**7200**	**9.8 × 10^−8^**
**Polyurethane**	**0.135**	**5.6**	**120**	**1.65 × 10^−8^**
